# Basic Fibroblast Growth Factor Activates MEK/ERK Cell Signaling Pathway and Stimulates the Proliferation of Chicken Primordial Germ Cells

**DOI:** 10.1371/journal.pone.0012968

**Published:** 2010-09-23

**Authors:** Jin Won Choi, Sujung Kim, Tae Min Kim, Young Min Kim, Hee Won Seo, Tae Sub Park, Jae-Wook Jeong, Gwonhwa Song, Jae Yong Han

**Affiliations:** 1 WCU Biomodulation Major, Department of Agricultural Biotechnology, Seoul National University, Seoul, Korea; 2 Avicore Biotechnology Institute, Optifarm Solution Inc., Gyeonggi-do, Korea; 3 Department of Molecular and Cellular Biology, Baylor College of Medicine, Houston, Texas, United States of America; Temasek Life Sciences Laboratory, Singapore

## Abstract

**Background:**

Long-term maintenance of avian primordial germ cells (PGCs) *in vitro* has tremendous potential because it can be used to deepen our understanding of the biology of PGCs. A transgenic bioreactor based on the unique migration of PGCs toward the recipients' sex cord *via* the bloodstream and thereby creating a germline chimeric bird has many potential applications. However, the growth factors and the signaling pathway essential for inducing proliferation of chicken PGCs are unknown.

**Methodology/Principal Findings:**

Therefore, we conducted this study to investigate the effects of various combinations of growth factors on the survival and proliferation of PGCs under feeder-free conditions. We observed proliferation of PGCs in media containing bFGF. Subsequent characterization confirmed that the cultured PGCs maintained expression of PGC-specific markers, telomerase activity, normal migrational activity, and germline transmission. We also found that bFGF activates the mitogen-activated protein kinase kinase/extracellular-signal regulated kinase (MEK/ERK) signaling. Also, the expression of 133 transcripts was reversibly altered by bFGF withdrawal.

**Conclusions/Significance:**

Our results demonstrate that chicken PGCs can be maintained *in vitro* without any differentiation or dedifferentiation in feeder free culture conditions, and subsequent analysis revealed that bFGF is one of the key factors that enable proliferation of chicken PGCs *via* MEK/ERK signaling regulating downstream genes that may be important for PGC proliferation and survival.

## Introduction

Germ cells play important roles in species continuation by delivering genetic information to the next generation. In many animal species, they arise from a small population of cells known as primordial germ cells (PGCs) [1], [2], [3]. In chickens, PGCs are initially localized to the central zone of the *area pellucida* in stage X embryos [4]. They migrate to the germinal crescent at stage 4 (18–19 h after incubation) [5] and, between stages 10 and 12, move into blood vessels and begin circulating in the bloodstream [6], [7]. *Via* the circulatory system, PGCs finally migrate to the genital ridge [8], [9]. During migration, PGCs proliferate: about 30 PGCs are found in a stage X embryo, 200–250 in the germinal crescent [4], and more than 1,000 at stage 31 (7 days of incubation) [10].

The basic fibroblast growth factor (bFGF) is a member of the fibroblast growth factor family that plays diverse roles in regulating cell proliferation, migration, and differentiation during embryonic development [11], [12], [13]. In mammals, it appears to be important for self-renewal of human embryonic stem cells [14] and mouse spermatogonial stem cells (SSCs) [15]. FGF signaling is critical to PGC migration and thereby controls germ cell numbers in mice [16]. In chickens, bFGF is one of the factors supporting the proliferation of preblastodermal cells [17], embryonic germ cells (EGCs) [18], and PGCs [19]. However, it remains to be determined whether bFGF is essential for the proliferation of chicken PGCs.

Studies of the maintenance and proliferation of avian PGCs *in vitro* offer tremendous potential in understanding the physiology of PGCs and the production of transgenic bioreactors. A recent report showed that PGCs from the blood of stage 14–17 chicken embryos could be expanded when cultured on a feeder layer of Buffalo rat liver (BRL) cells or Sandoz inbred mouse-derived thioguanine-resistant and ouabain-resistant (STO) cells, in an undefined medium conditioned with BRL cells containing leukemia inhibitory factor (LIF), stem cell factor (SCF), and bFGF [19]. However, specific growth factors that are essential for PGC proliferation remain to be identified, and the complex and undefined parameters arising from the use of conditioned media have made the roles of individual growth factors impossible to evaluate.

In this report, we describe the development of a feeder-free PGC culture system that excludes the effects of undefined molecules from the feeder layer. By using this culture system, the effect of individual growth factors, including LIF, SCF, and bFGF, on the proliferation of chicken PGCs *in vitro* was evaluated.

## Results

### bFGF is Essential for The Proliferation of Chicken PGCs *in vitro*

**Figure 1:**
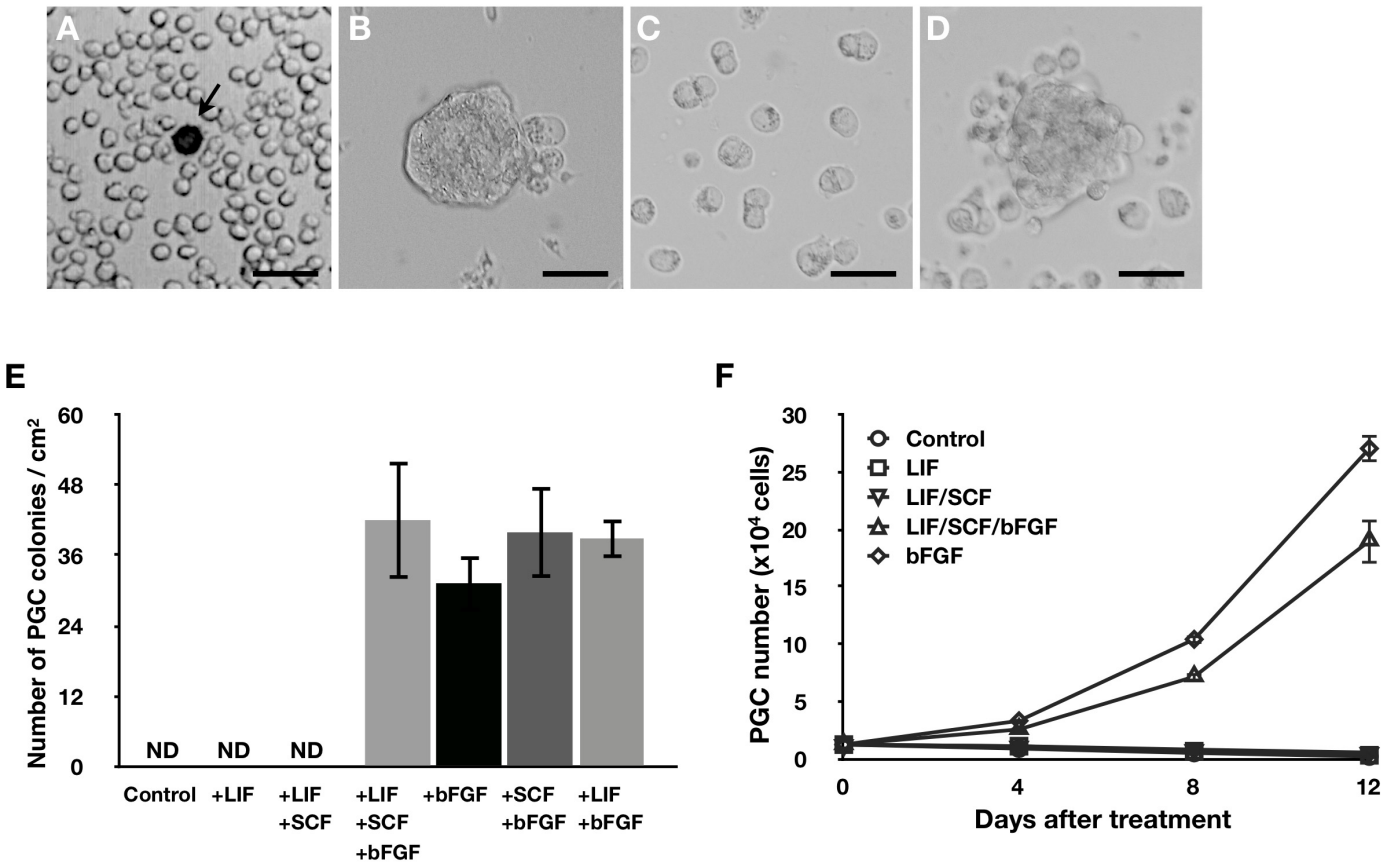
Culture of chicken PGCs. (A–D) Morphology of cultured PGCs. (A) Whole embryonic blood cells at day 0 in primary culture. PGCs were identified by SSEA-1 staining as indicated by an arrow. (B) PGC colonies after 10 days of culture. (C) Dissociated PGC colonies. (D) Reformation of PGC colonies (Bar  = 25 µm). (E) PGC colony formation following treatment with various combinations of growth factors (mean ± SEM; *n* = 9) (ND: not detected). (F) Growth curve of PGCs treated with different growth factors (mean ± SEM; *n* = 4).

Whole blood cells containing PGCs from chicken embryos at stage 14–15 were isolated (Fig. 1A) and cultured in the presence of LIF, SCF, and bFGF. After 7–14 days of growth, most of the blood cells were dead and PGC colonies formed and loosely attached to culture plate (Fig. 1B). The PGC colonies were detached by gentle pipetting and disaggregated with an appropriate enzyme (Fig. 1C). The disaggregated PGCs again grew and aggregated to form colonies in 3–4 days (Fig. 1D). The PGCs were then subcultured every 3–4 days. During subsequent passages, cultured PGCs did not attach to the culture plate and grew in suspension.

To identify the essential growth factors for PGC growth, we cultured PGCs under various combinations of growth factors. As illustrated in Fig. 1E, PGC colonies were only observed for cells grown in media containing bFGF. We next examined the effects of different growth conditions on PGC culture expansion. PGCs were cultured for 32 days (to passage 5) in media supplemented with LIF, SCF, and bFGF, and then continued to be cultured for a further 12 days under different conditions (Fig. 1F). When bFGF was absent from the culture medium, the number of cells decreased. In contrast, when bFGF alone or a combination of LIF, SCF, and bFGF was added to the culture medium, cell numbers increased at a constant rate over three passages. The results show that of the three growth factors tested, bFGF is essential for PGC proliferation.

**Figure 2:**
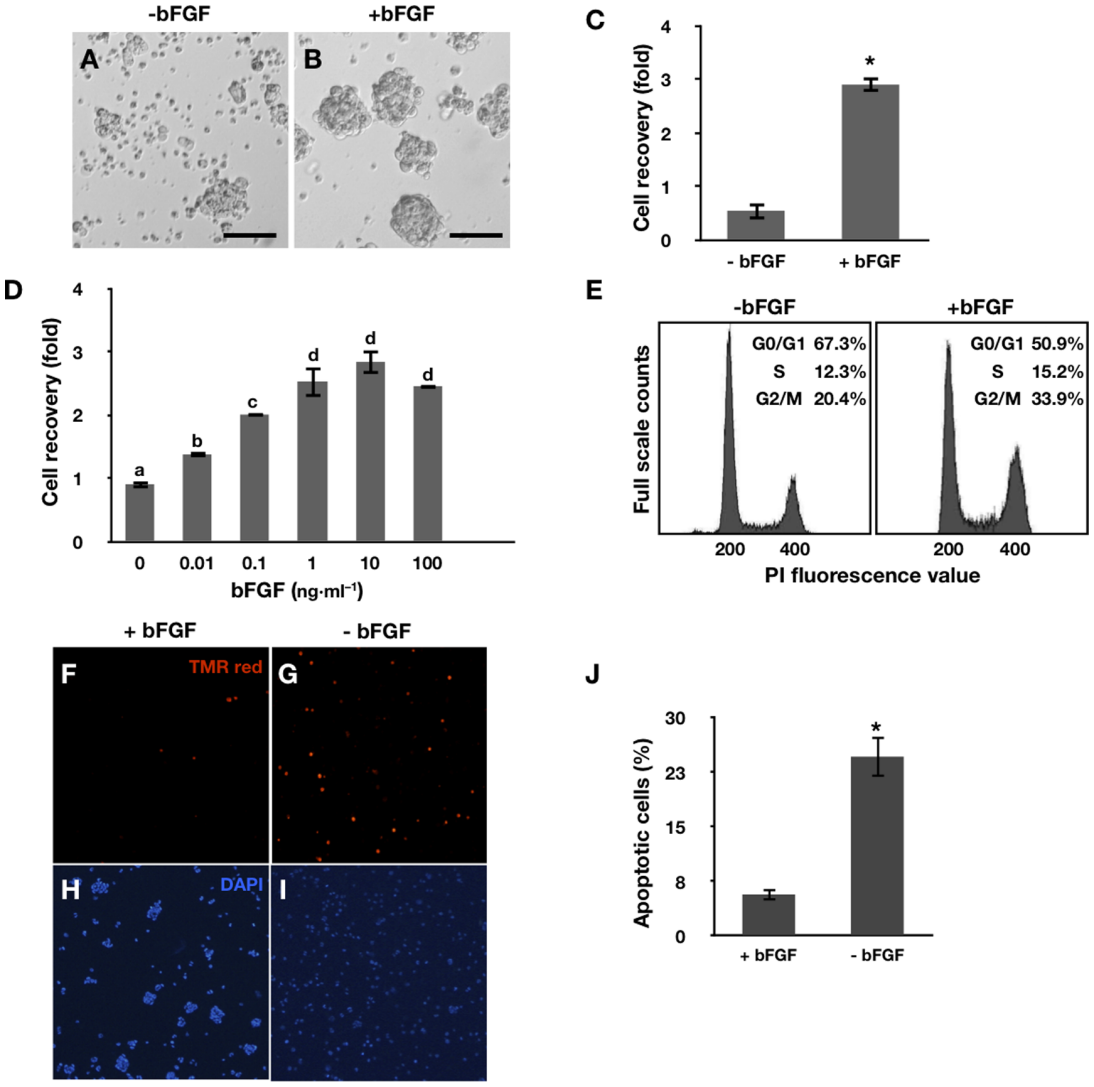
Effects of bFGF on PGC proliferation. (A–B) Morphology of PGCs in the presence of bFGF and 4 days after bFGF withdrawal (Bar  = 100 µm). (C) Effect of bFGF on cell recovery after 4 days of culture (mean ± SEM; *n* = 4) (*significant differences following treatment; *p*<0.01). (D) Dose-dependent effect of bFGF on the proliferation of PGCs (mean ± SEM; *n* = 3) (superscripts indicate significant differences between treatments; *p*<0.05). (E) Analysis of cell cycle distribution of PGCs in the presence of bFGF (left) and 24 h after bFGF withdrawal (right). (F–I) TUNEL assay performed on PGCs cultured with bFGF and 24 h after bFGF withdrawal (J) Number of apoptotic cells after bFGF withdrawal (*significant difference between treatments; *p*<0.01). Statistical analyses were conducted with a Student's *t*-test (C and J) or ANOVA using SAS software (D).

The effects of bFGF on PGC proliferation were examined in detail using cells that were cultured for more than 32 days. PGCs were cultured in the presence or absence of bFGF (10 ng·ml^−1^), and cell morphology and recovery were examined 4 days later. In the presence of bFGF, the proliferating PGCs formed colonies. In contrast, in the absence of bFGF, PGC growth was limited and a large number of fragmented cells were observed (Fig. 2A–2B). The total number of cells increased about threefold in the presence of bFGF, but decreased in the absence of bFGF (*p*<0.01; Fig. 2C). When PGCs were treated with different concentrations of bFGF, cell recovery increased in a dose-dependent manner (Fig. 2D). Low cell recovery in untreated cells may have resulted from increased apoptosis and reduced growth rates in the absence of bFGF. Thus, we next analyzed cell cycle progression and apoptosis of PGCs in the presence and absence of bFGF. Removal of bFGF for 24 h decreased the proportion of cells in the S and G2/M phase and increased the proportion of cells in the G1 phase (Fig. 2E). An increased number of apoptotic cells were observed by a TUNEL assay when bFGF was removed for 24 h (Fig. 2F–2J). Taken together, these results indicate that a single growth factor, bFGF, may support PGC proliferation. Therefore, we cultured PGCs in media containing bFGF and characterized them in detail.

### Characterization of Cultured Chicken PGCs

**Figure 3:**
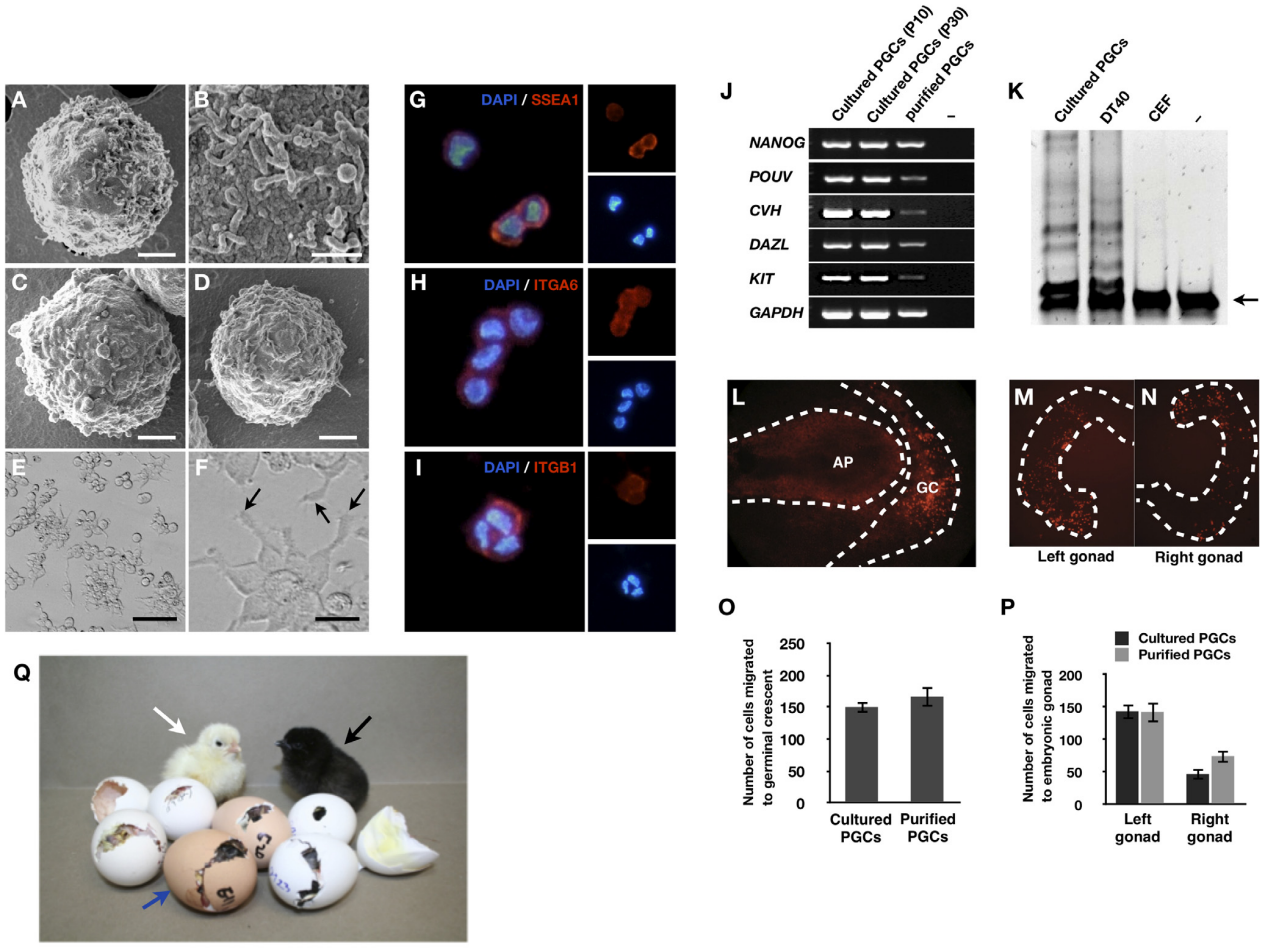
Characterization of cultured PGCs. Scanning electron microscopy of cultured PGCs (A–B), blood PGCs (C), and gonadal PGCs (D). (E, F) PGCs cultured on Matrigel (arrows indicate pseudopod-like structures). Bars: 2 µm (A, C, and D); 200 nm (B); 100 µm (E); and 25 µm (F). (G–I) Immunocytochemical analysis of cultured PGCs. PGCs cultured for 60 days were immunostained with antibodies raised against SSEA-1 (G), ITGA6 (H), and ITGB1 (I). (J) RT-PCR analysis of *NANOG*, *POUV*, *CVH*, *DAZL*, and *KIT* in cultured PGCs (passages 10 and 20) [–: negative control (no template)]. (K) Telomerase activity in PGCs. Repeated sequences were observed in PGCs and DT40 (positive control cells). Chicken embryonic fibroblast (CEF) and buffer (–) were used as negative controls. Arrow indicates the 56-bp internal control template band. (L) Migration of cultured PGCs into the germinal crescent. Approximately 500 PGCs, cultured for 82 days or purified by MACS, were labeled with PKH26 and then transferred into the subgerminal cavities of blastoderm embryos. Labeled cells (red) were detected in the germinal crescent (arrows) (GC: germinal crescent, AP: area pellucida). (M–N) Gonadal migration of culture PGCs. Approximately 500 PGCs, cultured for 82 days or purified by MACS, were labeled with PKH26 and then injected into blood vessels of recipient embryos at stage 14–17. Labeled cells (red) were detected in the embryonic gonad. (O) Numbers of PGCs that had migrated into the germinal crescent of stage 6 embryos that had, as stage X recipient embryos, been injected with 500 PGCs (mean ± SEM; *n* = 12 for purified PGCs, *n* = 11 for cultured PGCs). No significant difference was observed between cultured and purified PGCs. (P) Number of PGCs that had migrated into the gonads of stage 28 embryos that had, as stage 14 recipient embryos, been injected (i.v.) with 500 PGCs (mean ± SEM; *n* = 12 for purified PGCs, *n* = 10 for cultured PGCs). No significant difference was observed between cultured and purified PGCs. Statistical analysis was conducted with the general linear model (PROC-GLM) of SAS software. (Q) Germline transmission of cultured PGCs. Donor KO (*i*/*i*) PGCs cultured for more than 50 days were injected into the dorsal aorta of WL (*I*/*I*) recipient embryos (female), and after sexual maturation, progeny were derived from the donor KO PGCs (black plumage color, black arrow). Progeny that derived from the endogenous WL PGCs are noted by a white arrow. The white egg indicates that the recipient chicken is WL because KO chickens lay brown eggs as indicated by the blue arrow.

To observe detailed cell morphology, we visualized cultured PGCs (Fig. 3A–3B), PGCs purified from the blood of stage 14–17 embryos (Fig. 3C) and the gonad of stage 28 embryos (Fig. 3D) by scanning electron microscopy. All examined PGCs were approximately 9–12 µm in diameter and sphere-shaped with numerous microvilli. Moreover, when cultured on Matrigel, PGCs attached to the surface of the matrix. Pseudopodia-like structures were observed (Fig. 3E–3F).

Immunocytochemical analysis was performed to characterize cultured PGCs in detail. These cells were positive for the chicken PGC markers SSEA-1, ITGA6, and ITGB1 [20], [21] (Fig. 3G–3I) but negative for SSEA-3 and SSEA-4 (data not shown). To examine PGC-specific gene expression, RT-PCR analysis was performed (Fig. 3J). Expression of *NANOG* (NM_001146142.1) and *POUV* (NM_001110178), two genes known to be expressed in the germ cells of early-stage chicken embryos [22], were detected. The germ cell-specific genes *CVH* (NM_204708) [4], *DAZL* (NM_204218) [23], and *KIT* (NM_204361.1) [24] were also expressed in cultured PGCs. These same genes were similarly expressed in purified PGCs. Because a previous study showed that cultured PGCs have telomerase activity [19], we tested telomerase activity in PGCs cultured for 126 days. The result showed that the cultured PGCs used in our study also have telomerase activity (Fig. 3K). These data suggest that cultured PGCs are immortalized cells that maintain expression of both surface markers and PGC-specific genes.

Avian PGCs initially migrate to the germinal crescent before migrating to the genital ridge *via* the bloodstream [8], [25]. In addition, when injected into the dorsal aorta of stage 14–17 embryos, donor PGCs migrate to the gonads and contribute to the germ line [26], [27]. We therefore tested the migrational activity of cultured PGCs by two different strategies. First, PKH26-labeled PGCs that had been cultured for 82 days were injected into the subgerminal cavities of stage X blastoderm embryos that were then observed at stage 6 by fluorescence microscopy. Localization of the injected PGCs was restricted to the germinal crescent (Fig. 3L). Next, PKH26-labeled PGCs that had been cultured for 82 days were injected into the bloodstream of recipient stage 14–17 embryos that were subsequently observed at stage 30. Labeled cells were detected in the embryonic gonad (Fig. 3M–3N). Moreover, the number of cells that migrated did not significantly differ between cultured PGCs and PGCs purified from the gonad of stage 28 embryos when the they were injected into the subgerminal cavity of blastoderm embryos (*p* = 0.4759, Fig. 3O) and the dorsal aorta of stage 14–17 embryos (*p* = 0.5031, Fig. 3P). These results suggest that cultured PGCs have normal migrational activity.

When PGCs are injected into the blood vessels of recipient embryos during stages 13–17, germline chimera are produced [27], [28]. To confirm that the cultured PGCs can contribute to the germline, cultured PGCs (*i/i*) were injected into WL (*I/I*) recipient embryos. The putative germline chimeric chicken was sexually matured and donor-derived offspring with black plumage color were produced after artificial insemination with KO semen (Fig. 3Q and Table S1). To examine whether the cultured PGCs were dedifferentiated into EGCs during long-term culture, we also injected cultured PGCs into the subgerminal cavities of a stage X WL blastoderm, and 18 chicks subsequently hatched. However, we could not observe any somatic chimerism in the hatched chicks. These results confirm that the cultured PGCs are functionally normal.

### Activation of MEK/ERK Signaling by bFGF in PGCs

**Figure 4:**
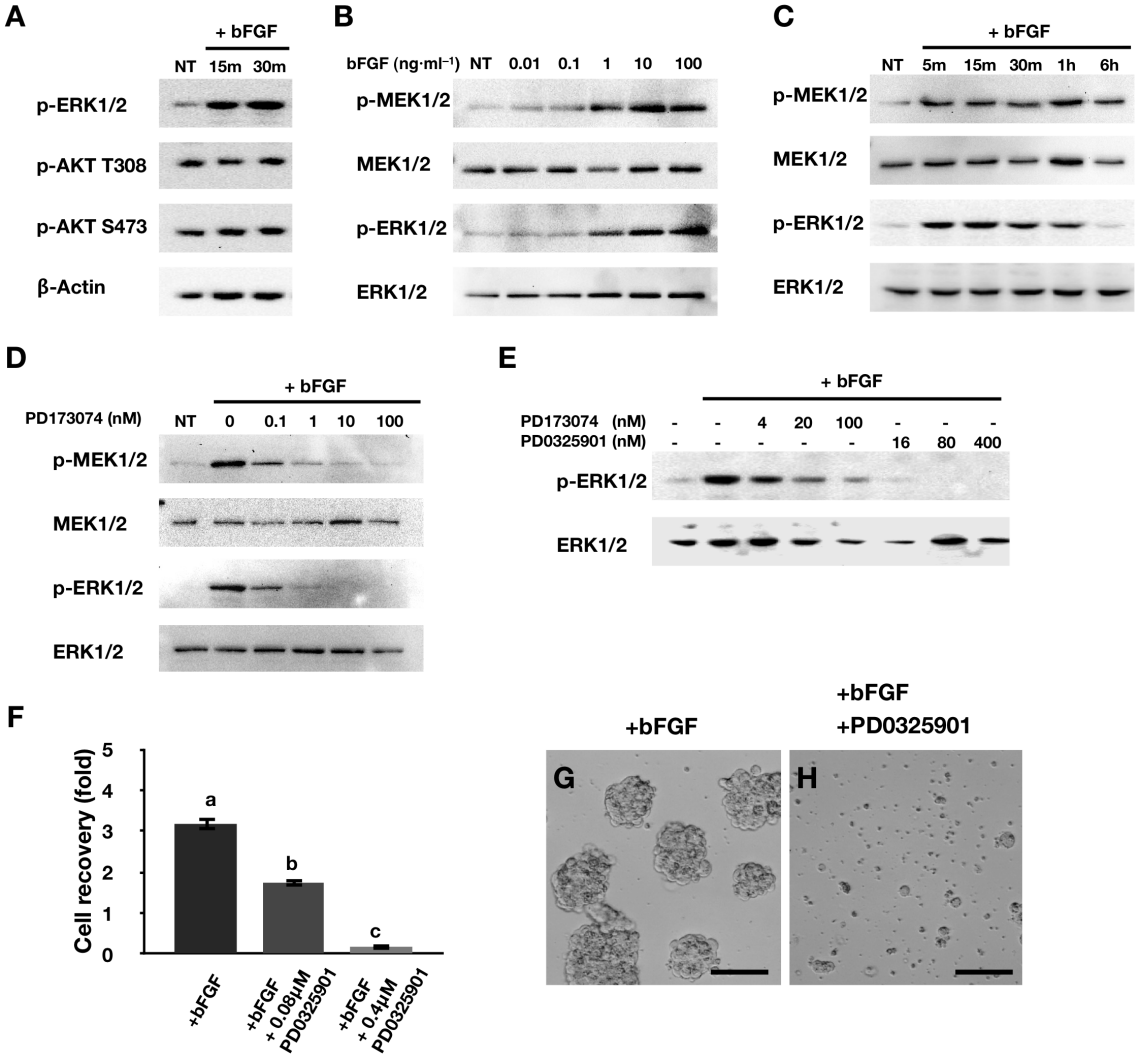
bFGF signaling in chicken PGCs. (A) Activation of MAPK and PI3K/AKT signaling pathways by bFGF (10 ng·ml^−1^). NT: no treatment (B) Dose-dependent activation of MEK/ERK signaling by bFGF. NT: no treatment (C) Temporal effect of bFGF (10 ng·ml^−1^) on activation of MEK/ERK signaling. NT: no treatment (D) Inhibition of MEK/ERK signaling by a specific inhibitor of FGFRs (PD173074). NT: no treatment (E) Inhibition of ERK activation by PD0325901 in cultured PGCs (PD173074 was used as a control). (F) Cell recovery 4 days after PD0325901 treatment (mean ± SEM, *n* = 4). bFGF (10 ng·ml^−1^) was treated in all groups. Superscripts indicate significant differences between treatment groups (*p*<0.05). Statistical analyses were conducted with ANOVA using SAS software. (G–H) Morphology of PGCs 4 days after treatment with PD0325901 (0.4 µM) (bar  = 100 µm). bFGF (10 ng·ml^−1^) was treated in all groups.

We examined the effect of bFGF on phosphorylation of AKT and three MAPKs: ERK1/2, p38, and JNK. PGCs were treated with bFGF, and the phosphorylation of MAPK and AKT was detected by a Western blot (Fig. 4A). Western blot analyses of whole PGC extracts using antibodies raised against p-(activated) target proteins showed that bFGF increased the level of p-ERK1/2 over the basal level. However, p-p38 and p-JNK MAPKs could not be detected (data not shown). AKT was phosphorylated on T380 and S473 independent of bFGF treatment.

We then examined the ERK1/2 signaling pathway in PGCs in more detail. In ERK signaling cascades, extracellular signals are transmitted by MEK1/2 [29]. Thus, we analyzed ERK1/2 signaling by simultaneously assessing the phosphorylation of both MEK1/2 and ERK1/2. Based on preliminary dose–response data, 10 ng·ml^−1^ bFGF was selected as the dose to be used in all experiments in the present study (Fig. 4B). As shown in Fig. 4C, bFGF stimulated increases in p-MEK1/2 and p-ERK1/2 levels within 15 min that were sustained for 6 h and 1 h, respectively. To determine the cell signaling pathways mediating the effects of bFGF on MEK1/2 and ERK1/2, PGCs were pretreated with a pharmacological inhibitor of FGFR (PD173074). Induction of p-MEK1/2 and p-ERK1/2 by bFGF was blocked by FGFR inhibition (Fig. 4D). These results suggest that MEK/ERK cascades are downstream targets of the FGF pathway in chicken PGCs. In addition, to examine whether inhibition of MEK/ERK signaling affects PGC survival, PGCs were treated with PD0325901, a specific inhibitor of MEK [30]. Activation of p-ERK1/2 by bFGF was dose-dependently inhibited by PD0325901 (Fig. 4E). Furthermore, PD0325901 significantly and dose-dependently reduced PGC recovery 4 days after bFGF treatment (*p*<0.05) (Fig. 4F). As shown in Fig. 4G–4H, most PGCs treated with PD0325901 were fragmented, and cell colonies were rare. Collectively, these results suggest that stimulation of PGC proliferation by bFGF requires activation of MEK1/2–ERK1/2 signaling.

### Effect of Withdrawal of bFGF for 24 h on the Potential of PGCs

**Figure 5:**
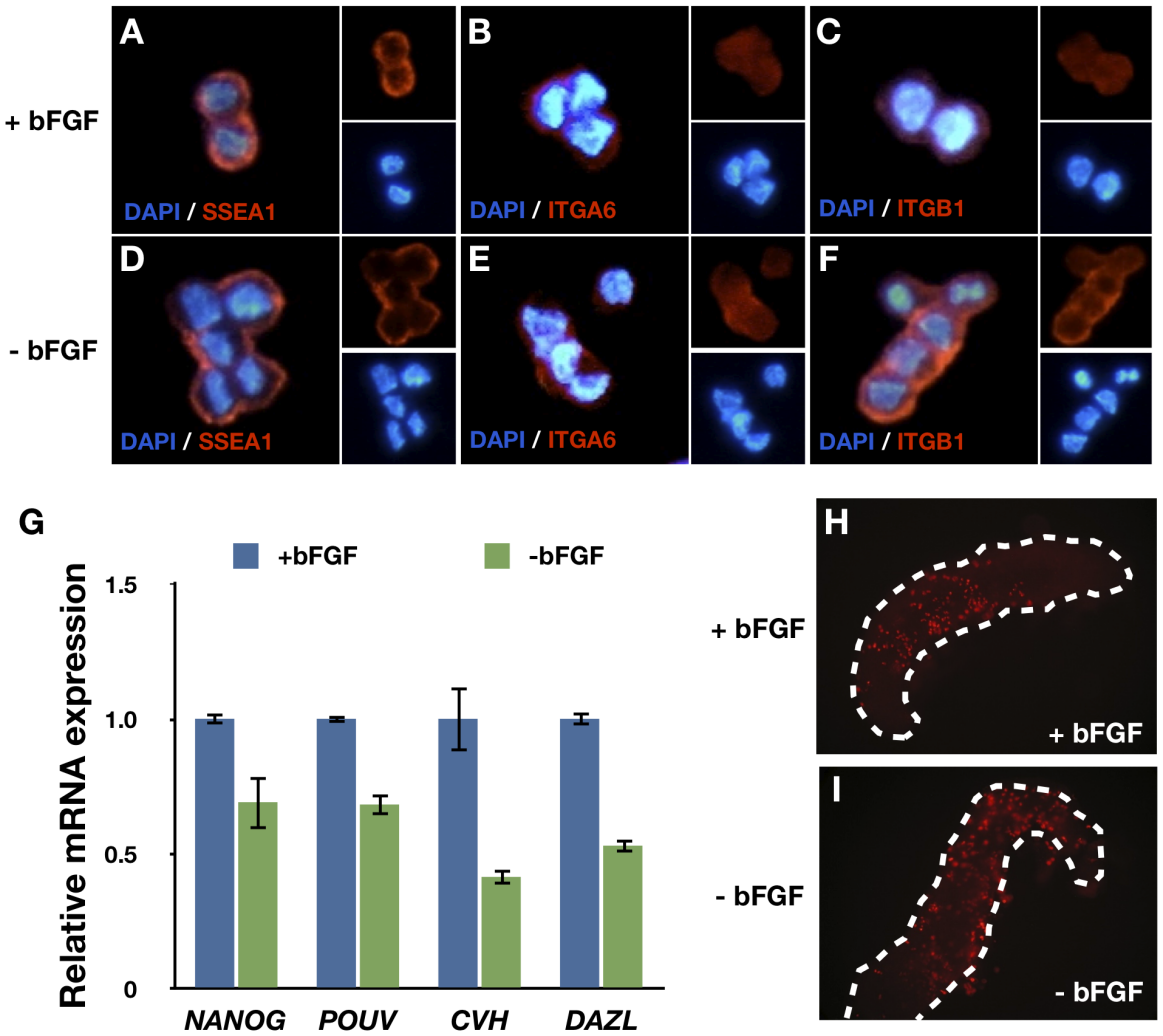
Effect of bFGF withdrawal on the potential of PGC. bFGF withdrawal did not significantly affect the potential of PGC. (A–F) Immunocytochemical analysis of cultured PGCs 24 h after bFGF withdrawal. (G) Gene expression analysis of cultured PGCs 24 h after bFGF withdrawal (mean ± SEM; *n* = 3). (H–I) Migrational activity of PGCs 24 h after bFGF withdrawal.

To examine the effect of bFGF on the potential of PGCs, PGCs were characterized following withdrawal of bFGF for 24 h. Antibody staining showed that bFGF withdrawal did not affect the expression of the markers SSEA-1, ITGA6, and ITGB1 (Fig. 5A–5F). In contrast, quantitative RT-PCR analysis demonstrated that expression of PGC marker genes including *NANOG*, *POUV*, *CVH*, and *DAZL* was downregulated following bFGF withdrawal (Fig. 5G). However, injection of PGCs into the blood vessels of recipient embryos revealed that their migrational activity was not significantly affected by bFGF withdrawal (Fig. 5H). These data suggest that the withdrawal of bFGF for 24 h does not significantly affect the potential of PGC.

### Identification of bFGF-Regulated Genes in PGCs

**Figure 6:**
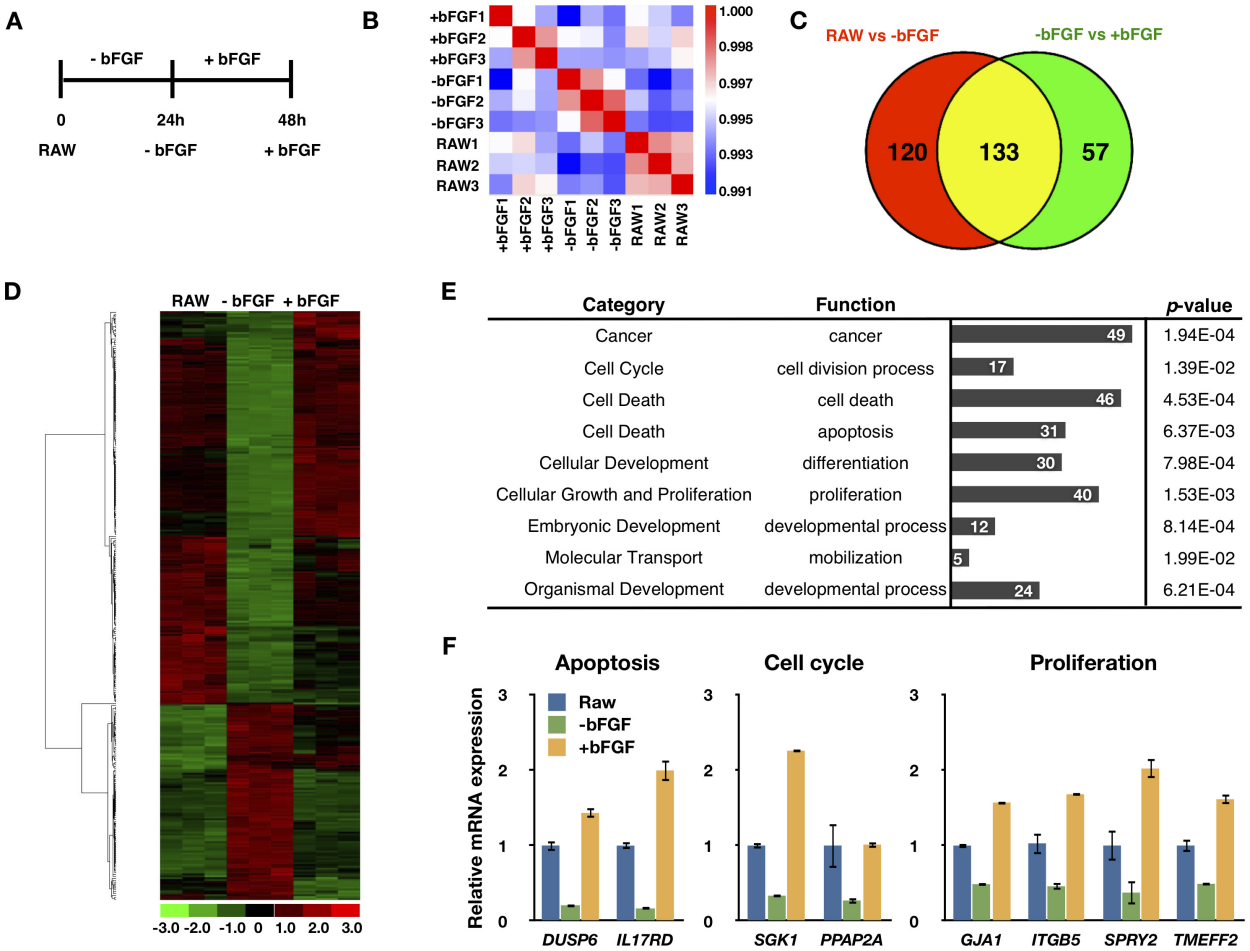
Identification of bFGF-regulated transcripts in PGCs. (A) Schematic representation of sample preparation. RNA samples were extracted at three time points: before bFGF withdrawal (RAW), 24 h after bFGF withdrawal (-bFGF), and 24 h after bFGF replacement (+bFGF). (B) Pearson's correlation matrix of microarray data. (C) Venn diagram distribution of bFGF-regulated genes. (D) Hierarchical clustering of bFGF-regulated transcripts. Clustering was performed on 322 transcripts whose expression changed at least 1.2-fold among the three groups. (E) Functional categorization of genes whose expression changed following bFGF withdrawal and replacement. (F) Validation by quantitative RT-PCR of the microarray data relating to genes with roles in the control of apoptosis, cell cycle, and proliferation (mean ± SEM; *n* = 3).

To identify transcriptional genes regulated by bFGF in chicken PGCs, we cultured chicken PGCs without bFGF for 24 h (-bFGF) and re-added bFGF for another 24 h (+bFGF). We compared the gene transcription profiles between PGCs before bFGF withdrawal (RAW, 0 h), without bFGF (-bFGF, 24 h) and re-adding of bFGF (+bFGF, 48 h) through the microarray analysis (Fig. 6A). In microarray analyses, a correlation matrix showed the between-group variation to be greater than the within-group variation (Fig. 6B). Following bFGF withdrawal, 162 transcripts were downregulated and 91 upregulated. When bFGF was replaced, 132 transcripts were upregulated and 58 downregulated (Table S2). In total, we identified 310 transcripts whose expression was changed at least 1.2-fold by bFGF withdrawal or replacement. Moreover, of the gene expression changes that followed bFGF withdrawal, 133 were reversed by bFGF replacement (Fig. 6C). Hierarchical clustering analysis also showed that most of the gene expression changes that followed bFGF withdrawal were reversed by bFGF replacement, though in some cases only partially (Fig. 6D).

We next categorized the bFGF-regulated genes into specific functional groups according to gene ontology (GO). GO groups that were enriched in the lists of genes whose expression was altered by bFGF withdrawal or replacement included cancer, cell division process, cell death, apoptosis, differentiation, proliferation, developmental process, and mobilization (Fig. 6E and Table S3). The microarray results showed that many genes involved in cell survival and proliferation were regulated by bFGF. These include *SPRY2* (NM_204800.1), *PPAP2A* (XM_424730.2), *GJA1* (NM_204586.1), and *TMEFF2* (XM_001231528.1), all of which are involved in cell cycle or proliferation and *IL17RD* (NM_204515.1), *DUSP6* (NM_204354.1), *SGK1* (NM_204476.1), and *ITGB5* (NM_204483.1), which are classified as cell death genes (Table S4). We validated the microarray data by quantitative RT-PCR. The results showed agreement between the microarray expression profile and quantitative RT-PCR data (Fig. 6F). Collectively, these data suggest that bFGF's regulation of genes with roles in the control of cell proliferation and survival may promote PGC population expansion *in vitro*.

## Discussion

Specific growth factors and feeder cells are reportedly required for the culture of PGCs *in vitro*. In zebrafish, epidermal growth factor, bFGF, Kit ligand-a, stromal cell-derived factor-1b, and RTS34st feeder cells were used to culture PGCs [31]. In mice and humans, three growth factors, namely LIF, SCF, and bFGF, and STO feeder cells are required for the culture of EGCs [32], [33]. Chicken PGCs have been cultured in media supplemented with the same growth factors (except for LIF, which is produced from BRL-conditioned media) and feeder cells as mammalian EGCs [19]. In contrast to mammalian EGCs and zebrafish PGCs, which attach to the culture surface, the chicken PGCs in our system grew in suspension without physical interaction with the feeder layer. We therefore reasoned that the major role of the feeder layer might be to supply growth factors, and hypothesized that the addition of essential growth factors to the culture medium might be enough to support *in vitro* proliferation of PGCs. To test our hypothesis, PGCs were cultured in media supplemented with different growth factors under feeder-free condition. Moreover, we did not use BRL-conditioned media. As a result, we identified bFGF as being an essential factor for *in vitro* PGC proliferation because PGCs grew in media supplemented with bFGF alone and maintained PGC characteristics. In mammals, bFGF also acts as a mitogen for PGCs *in vitro* [33]. However, a recent study showed that bFGF is also a key factor in reprogramming PGCs to become EGCs [34]. bFGF downregulates *Blimp1* (which plays a critical role in the specification and maintenance of the early germ cell lineage [35]) resulting in upregulation of *Blimp1* targets including *c-Myc* and *Klf4*, which are the key factors in promoting reprogramming somatic cells to become pluripotent [36]. However, we did not, in the present study, observe EGC-like colonies during culture of more than 150 days.

van de Lavoir et al. [19] reported in vitro culture of chicken PGCs and they used STO cells as a feeder and BRL-conditioned media. But in our culture condition, chicken PGCs can be maintained without feeder layer (a feeder-free) and BRL-conditioned media. In this paper, we found that FGF signaling is more essential than a feeder or BRL-conditioned media for in vitro proliferation of chicken PGCs.

PGCs grew to form cell colonies under the growth conditions we used, which contradicts a previous report in which PGCs grew as single cells [19]. Growth factors may not be the major cause of the colony formation as PGCs formed colonies with all growth factor combinations we tested. This discrepancy possibly stems from the other differences in the two different culture methods for PGCs. The essential differences are the use of feeder layers and BRL-conditioned media. Further study will be needed to reveal what factors cause PGCs to form colonies. However, colony formation may be normal. A study in mice found that PGCs aggregate together and that interactions between PGCs play a role in the accumulation of PGCs in the genital ridge [37]. In chickens, PGCs were previously shown to aggregate to form cell colonies not attaching to the surface provided during culture [38].

Morphological analysis of PGCs in a previous report demonstrated that PGCs were sphere-shaped with numerous microvilli [39]. Moreover, it has been reported than when PGCs adhered to a collagen layer, the majority of the PGCs produced small pseudopodia [40]. For our data, PGCs also had numerous microvilli and generated pseudopodia when attached to Matrigel. These results suggest that cultured PGCs maintain similar morphological characters to those of PGCs *in vivo*.

In the present study, PGCs expressed SSEA-1, ITGA6, and ITGB1 but not SSEA-3 or SSEA-4. In a previous study, we reported that SSEA-3 and SSEA-4 were markers for PGCs and EGCs, assuming that PGCs were cultured for fewer than 10 days to maintain the characteristics of PGCs [20]. This disparity could arise from the different culture conditions employed that may have resulted in different glycochain expression. Alternatively, the PGCs might have differentiated into EGCs within 10 days of culture in the previous study. Indeed, a recent study in mice showed that differentiation of PGCs into EGCs takes approximately 10 days [34].

Migrational activity is a key characteristic of PGCs. In stage X blastoderms, about 30 PGCs are scattered in the central zone of the *area pellucida* [4]. After segregation from the epiblast, PGCs are known to passively relocate to the germinal crescent during gastrulation [25], [41]. Although we were unable to define the exact mechanism by which PGCs translocate to the germinal crescent, our results somewhat support the notion that PGCs move actively to the germinal crescent as most of the exogenous PGCs that had been introduced localized to the germinal crescent. Had they moved passively, injected PGCs would be expected to be distributed evenly throughout other areas.

Many signaling pathways may support the biology of stem cells. In mouse embryonic stem cells (ESCs), signaling *via* LIF/STAT3 [42], BMP/ID [43], PI3K/AKT [44], [45], and Src [46] plays a critical role in maintaining the capacity for self-renewal. In human ESCs, MEK/ERK signaling, which lies downstream of the FGF receptor, is required for the maintenance of pluripotency, while PI3K/AKT signaling regulates cell proliferation and survival [47]. In mouse SSCs, glial cell-derived neurotrophic factor (GDNF) activates downstream signals that mediate self-renewal *via* the PI3K/AKT pathway [48] and also promotes mSSC proliferation by upregulating c-Fos transcription *via* the ERK/MEK pathway [49]. In the present study, we examined MAPK and PI3K/AKT signaling molecules as candidate downstream effectors of bFGF responses as FGFR is a receptor tyrosine kinase whose activation induces cell proliferation and differentiation *via* the MEK/ERK pathway or PI3K/AKT pathway during early development in vertebrates [50]. Two other MAPK pathways, p38 and JNK, were also found to be activated by FGF signaling in different cell types [51], [52], [53]. Our data collectively show that MEK/ERK is a downstream target of bFGF that activates a diverse range of second messengers and supports cell proliferation in chicken PGCs.

Although bFGF stimulates MEK/ERK signaling and induces the proliferation of PGCs, MEK/ERK alone was not enough to prevent PGCs from losing their unique potential because bFGF withdrawal did not significantly alter marker expression or migrational activity. In addition to MEK/ERK signaling, other signaling pathways may play important roles in maintaining PGC characteristics.

We also identified genes whose expression in PGCs was altered by bFGF. Of them, *IL17RD* was the most markedly changed gene (by both bFGF withdrawal and replacement). *IL17RD* (also known as *SEF*) is a spatial regulator of RAS/MAPK signaling. SEF specifically blocks ERK nuclear translocation without inhibiting its activity in the cytoplasm, consequently inhibiting phosphorylation and activation of the nuclear ERK substrate, ELK-1 [54]. Therefore, MEK/ERK signaling may activate cytoplasmic substrates such as RSK2. The expression profiles of two negative feedback regulators of MEK/ERK, *SPRY2* and *DUSP6* (also known as *MKP3* [50]) were similar to that of *IL17RD*, suggesting that excessive activation of ERK adversely affects the potential of PGC. The alteration in *DUSP6* expression may also relate to our observation that phosphorylation of ERK1/2 decreased 6 h after bFGF treatment. In addition, the bFGF-regulated genes included several with cellular functions important for cell survival and proliferation, including ion transport (*SGK1*) [55], glycolipid metabolism (*PPAP2A*) [56], and cell proliferation [57]. However, functional studies are needed to confirm their precise functions in PGCs.

We conclude that bFGF is an essential growth factor for the *in vitro* culture of chicken PGCs under feeder-free conditions, whose effects are mediated by MEK/ERK. Our results provide novel insights into the physiology of germ cells in other species as chickens are the only vertebrate in which unlimited expansion of PGCs is feasible. PGCs may become a versatile tool for generating transgenic bioreactors and avian models.

## Materials and Methods

### Experimental Animals and Animal Care

The care and experimental use of chickens were approved by the Institute of Laboratory Animal Resources, Seoul National University (SNU-070823-5). Korean Oge (KO) and White Leghorn (WL) chickens were maintained according to a standard management program at the University Animal Farm, Seoul National University, Korea. The procedures for animal management, reproduction, and embryo manipulation adhered to the standard operating protocols of our laboratory.

### Culture of PGCs

Approximately 2 µl of whole blood cells taken from the dorsal aorta of stage 14–15 (H&H) (50–54 h of incubation) KO chicken embryos (mixed-sex) were mixed and cultured in media comprising knockout Dulbecco's modified Eagle's medium (Invitrogen, Carlsbad, CA), 7.5% fetal bovine serum (Hyclone, Logan, UT), 2.5% chicken serum (Sigma-Aldrich, St. Louis, MO), 2 mM GlutaMAX-I Supplement (Invitrogen), 1× nucleosides (Millipore, Temecula, CA), 1× nonessential amino acids, β-mercaptoethanol, and combinations of the following growth factors: 2 ng·ml^−1^ human LIF (Sigma-Aldrich), 5 ng·ml^−1^ human SCF (Sigma-Aldrich), and 10 ng·ml^−1^ human bFGF (Sigma-Aldrich). Cells were cultured in a CO_2_ incubator maintained at 37°C in an atmosphere of 5% CO_2_ in air with 60%–70% relative humidity. The cultured PGCs were subcultured at 3- to 4-day intervals by dissociating cell colonies using Accutase (Millipore). To generate pseudopodia, PGCs were cultured on hESC-qualified Matrigel (BD Biosciences, San Jose, CA).

### Cell cycle analysis

Cultured cells were fixed with 70% ethanol at 4°C overnight and incubated with RNase (100 µg·ml^−1^; Sigma-Aldrich) for 5 min. After addition of propidium iodide (50 µg·ml^−1^ in PBS; Sigma-Aldrich), DNA content was analyzed using a FACS Calibur flow cytometer (BD Biosciences).

### TUNEL Assay

Cells were washed and concentrated on glass slides. After fixation in a formalin-ethanol fixative (4% formalin in 95% ethanol) for 10 min, the cells were incubated in a permeabilization solution (0.1% Triton X-100 in PBS). Apoptotic cells were identified using an In Situ Cell Death Detection Kit, TMR red (Roche Applied Science, Basel, Switzerland) that stains apoptotic cells red. Cells were counterstained with DAPI, mounted, and analyzed under a fluorescence microscope.

### Scanning Electron Microscopy

Cultured PGCs were fixed in 2% glutaraldehyde in 0.1 M sodium cacodylate buffer (SCB; pH 7.2) and post-fixed in 1% osmium tetroxide in SCB at 4°C for 2 h. After dehydration in a graded series of increasing concentrations of ethanol, the samples were immersed in hexamethyldisilazane and then dried. The samples were observed using a SUPRA 55VP field emission scanning electron microscope (Carl Zeiss, Oberkochen, Germany) at the National Instrumentation Center for Environmental Management (NICEM) at Seoul National University.

### Immunocytochemistry

A protocol adapted from a previous report [20] was used for immunocytochemistry. Briefly, cultured PGCs were fixed in 3.7% paraformaldehyde solution for 10 min, washed three times with phosphate-buffered saline (PBS) and blocked with blocking buffer, consisting of PBS containing 5% (v/v) goat serum and 1% bovine serum albumin, for 30 min and then incubated with primary antibodies diluted 1∶200 in blocking buffer at 4°C overnight. Primary antibodies raised against SSEA-1 (Santa Cruz Biotechnology, Santa Cruz, CA), SSEA-3 (Santa Cruz Biotechnology), SSEA-4 (Santa Cruz Biotechnology), ITGA6 (Millipore), and ITGB1 (Millipore) were used. Following three washes with PBS, cells were incubated with secondary antibodies labeled with phycoerythrin or fluorescein isothiocynate (Santa Cruz Biotechnology) for 1 h at room temperature. Cells were finally mounted with ProLong® Gold antifade reagent (with DAPI, or 4′,6-diamidino-2-phenylindole) (Invitrogen) and analyzed under a fluorescence microscope. For PGCs in whole embryonic blood at day 0, staining was carried out using the SSEA-1 antibody and DAKO Universal LASB® kit, Peroxidase (DAKO, Carpinteria, CA) according to the manufacture's instruction.

### RT-PCR Analysis

Total RNA samples were prepared using an RNeasy Mini Kit (Qiagen, Valencia, CA) and cDNA synthesized using AccuPower® RT PreMix (Bioneer, Daejeon, Korea). RT-PCR was performed using specific primer sets (Table S5). Reactions comprised 35 cycles at 95°C for 20 s, 60°C for 40 s, and 72°C for 1 min. RNA was extracted from purified PGCs that were isolated by magnetic-activated cell sorting (MACS) [58] from gonadal cells from stage 27–28 chicken embryos using an anti-SSEA-1 antibody, and this RNA was used as a control. Primer set information is listed in Table S5.

### Detection of Telomerase Activity

Cultured PGCs were pelleted and frozen at −70°C before analysis. Telomerase activity was detected using a TRAPEZE® XL Telomerase Detection Kit (Millipore) according to the manufacturer's protocol. DT40 was used as a positive control, and chicken embryonic fibroblasts (CEFs) and lysis buffer as negative controls.

### Migration Assay

PGCs cultured for 82 days were used for the migration assay. To assay migration into the germinal crescent, cultured PGCs were labeled with PKH26 fluorescent dye (Sigma-Aldrich) and then transferred into the subgerminal cavity of stage X embryos. After they were sealed with Parafilm, eggs were further incubated until stage 6 (24 h of incubation). Embryos were cut away from the yolk with the aid of filter paper and microdissecting scissors and then the number of fluorescent PGCs in the germinal crescent of excised embryos was counted under a fluorescence microscope (IX-70, Olympus, Tokyo, Japan). To assay migration into the gonads, PKH26-labeled PGCs were injected into the dorsal aorta of stage 14–17 embryos. After they were sealed with Parafilm, eggs were further incubated until stage 30. Gonads from the recipient embryos were retrieved, and then the number of fluorescent PGCs in the gonad was counted under a fluorescence microscope (IX-70, Olympus).

### Production of Germline Chimeric Chicken

PGCs cultured for more than 50 days were used for germ cell transfer. A small window was made at the pointed end of the recipient egg, and 2 µl (containing approximately 3,000 cells) was injected into the upper portion of the dorsal aorta of the stage 13 WL embryo (50 h of incubation) using a micropipette. The window was then sealed twice with Parafilm, and the egg was incubated with the pointed end down until hatching. Putative germline chimeric chickens that reached sexual maturity were then testcrossed by mating with KO (*i/i*) chickens of the opposite sex. Donor PGC-derived offspring could be identified based on their color: donor PGC-derived progeny (*i/i*) had black feathers, whereas the progeny (*I/i*) from endogenous WL PGC (*I/I*) had white feathers.

### Western Blot Analysis

Total protein was extracted using a Qproteome™ Mammalian Protein Prep Kit, separated on a 10% polyacrylamide gel and then transferred to a polyvinylidene fluoride membrane (Millipore). The following primary antibodies were used: rabbit anti-ERK1/2, anti-phospho (p)-ERK1/2, anti-MEK1/2, anti-p-MEK1/2, anti-AKT, anti-p-AKT (Thr308), and anti-p-AKT (Ser473) (Cell Signaling Technology, Danvers, MA); and mouse anti-β-actin (Santa Cruz Biotechnology). Peroxidase-conjugated anti-rabbit IgG and anti-mouse IgG (Santa Cruz Biotechnology) were used as secondary antibodies. The blots were treated with the ECL substrate solutions (AbFrontier, Seoul, Korea) and exposed in a ChemiDoc XRS System (Bio-Rad Laboratories, Hercules, CA) to detect chemiluminescence.

### Microarray

Microarray analysis was performed using Affymetrix GeneChip® Chicken Genome Arrays (Affymetrix, Santa Clara, CA). Data were generated by the Seoulin Bioscience Cooperation (Seoul, Korea). Total RNA was extracted from three different treated cell samples using an RNeasy Total RNA Isolation Kit (Qiagen). All experiments were repeated three times. Briefly, we used dChip software [59]. We selected differentially expressed genes at each time point by two-sample comparisons using the following criteria: lower boundary of a 90% confidence interval for fold-changes greater than 1.2, and absolute value of differences between group means greater than 50. Differentially regulated genes identified in the microarray analyses were analyzed using Ingenuity Pathways Analysis software (Ingenuity Systems, Mountain View, CA). Canonical pathway analyses identified the pathways from the Ingenuity Pathways Analysis library of canonical pathways that were most highly represented in the data set. The raw data has been deposited in a MIAME compliant format in the GEO database, accession number GSE22592.

### Quantitative RT-PCR

Total RNA samples were prepared using RNeasy Mini Kits (Qiagen) and cDNA was synthesized using AccuPower® RT PreMix (Bioneer). Gene expression levels were measured using Evagreen® dye (Biotium Inc., Hayward, CA) and a CFX96 Real-Time PCR Detection System (Bio-Rad Laboratories). Quantification of relative gene expression was calculated using the following formula: 2^−ΔΔCt^, where ΔΔCt = (Ct of the target gene - Ct of *GAPDH*)_treatment_ -(Ct of the target gene - Ct of *GAPDH*)_control_. Primer set information is listed in Table S6.

### Statistical Analysis

Effects of bFGF on cell recovery and apoptosis were analyzed with a Student's *t*-test. To determine if any significant differences in recovery existed between cells treated with different concentrations of bFGF and PD0325901, an analysis of variance (ANOVA) was performed using SAS software (SAS Institute, Cary, NC). To determine if any significant differences existed between cultured PGCs and purified PGCs in the migration assay, the data were analyzed with the general linear model (PROC-GLM) of SAS software. If the main effect was significant, the effects of individual treatments were compared by the least-significant difference (LSD) method. A *p* value of less than 0.05 was considered to be statistically significant.

## Supporting Information

Table S1 Progeny test of donor-derived chicks from putative germline chimeric chickens produced by transplantation of cultured PGCs. ^a^Portion of all offspring that were donor-derived (*i*/*i*). Values in parentheses are percentages.
(0.03 MB DOC)

Table S2 Number of genes changed in each comparison of the treatments in the microarray analysis.
(0.03 MB DOC)

Table S3 Categorization of the bFGF-regulated genes into specific functional groups by gene ontology terms.
(0.05 MB DOC)

Table S4 Regulation of cell proliferation and survival genes by bFGF. ^a^From microarray data from three samples of cultured PGCs after bFGF withdrawal. ^b^From microarray data from three samples of cultured PGCs after bFGF replacement.
(0.09 MB DOC)

Table S5 Information of the primer sets used for RT-PCR analysis.
(0.03 MB DOC)

Table S6 Information of the primer sets used for quantitative RT-PCR analysis.
(0.04 MB DOC)
