# Supplementary material for: Basic Fibroblast Growth Factor Activates MEK/ERK Cell Signaling Pathway and Stimulates the Proliferation of Chicken Primordial Germ Cells
Source: PLoS One. 2010 Sep 23;5(9):e12968. doi: 10.1371/journal.pone.0012968 (PMC2944891; doi:10.1371/journal.pone.0012968)
Supplement: Table S1 — Progeny test of donor-derived chicks from putative germline chimeric chickens produced by transplantation of cultured PGCs. aPortion of all offspring that were donor-derived (i/i). Values in parentheses are percentages. (0.03 MB DOC) [file pone.0012968.s001.doc]

| ID of Putative germline chimeras | Sex | No. of hatched chicks | No. of donor-  derived chick  produced (%)^a^ |
| --- | --- | --- | --- |
| 723 | Female | 69 | 57 (82.6) |
| 750 | Male | 13 | 7 (53.8) |
| 753 | Female | 8 | 1 (12.5) |
| 754 | Female | 50 | 21 (42.0) |
| 1210 | Female | 46 | 25 (54.3) |
