# Supplementary material for: Basic Fibroblast Growth Factor Activates MEK/ERK Cell Signaling Pathway and Stimulates the Proliferation of Chicken Primordial Germ Cells
Source: PLoS One. 2010 Sep 23;5(9):e12968. doi: 10.1371/journal.pone.0012968 (PMC2944891; doi:10.1371/journal.pone.0012968)
Supplement: Table S2 — Number of genes changed in each comparison of the treatments in the microarray analysis. (0.03 MB DOC) [file pone.0012968.s002.doc]

| Comparison | No. of down-regulated genes | No. of up-regulated genes | Total No. of genes |
| --- | --- | --- | --- |
| RAW vs. -bFGF | 162 | 91 | 253 |
| -bFGF vs. +bFGF | 58 | 132 | 190 |
| RAW vs. +bFGF | 23 | 28 | 51 |
