# Supplementary material for: Basic Fibroblast Growth Factor Activates MEK/ERK Cell Signaling Pathway and Stimulates the Proliferation of Chicken Primordial Germ Cells
Source: PLoS One. 2010 Sep 23;5(9):e12968. doi: 10.1371/journal.pone.0012968 (PMC2944891; doi:10.1371/journal.pone.0012968)
Supplement: Table S3 — Categorization of the bFGF-regulated genes into specific functional groups by gene ontology terms. (0.05 MB DOC) [file pone.0012968.s003.doc]

| Category | Function | *p*-value | Molecules | No. of Molecules |
| --- | --- | --- | --- | --- |
| Cancer | cancer | 1.94E-04 | AKAP12, ANXA2, BARD1, BLNK, CA2, CA9, CCND1, CCND2, CD82, CMTM8, COL1A2, CXCL12, CXCR4, DHCR24, DR1, DUSP6, ETV5, FDFT1, FECH, GFRA1, GJA1, HMGB1 (includes EG:3146), IDI1, ITGB5, KCNG1, LDHA, MSH6, MYCN, NEFM, NLGN1, NRIP1, P4HA1, PCP4, PDGFA, PITX2, PPAP2A, PTGR1, RHOB, SC4MOL, SERPINE2, SFRP2, SGK1, SLC2A3, SLC34A2, SMAD2, SMARCA2, SQLE, TRIM24, TSC1 | 49 |
| Cell Cycle | cell division process | 1.39E-02 | AGK, AKAP12, BLNK, BUB1, CCND1, CCND2, CD38, CXCL12, GFRA1, GJA1, GNAI1, MYCN, PITX2, PLK2, PPAP2A, SMARCA2, SPRY2 | 17 |
| Cell Death | cell death | 4.53E-04 | AKAP12, ATP2B1, BARD1, BLNK, BUB1, CADM1, CCND1, CCND2, CD38, CD82, CDC37, CTBP2, CTH, CXCL12, CXCR4, DHCR24, DUSP4, DUSP6, EAF2, EOMES, EZR, FDFT1, GFRA1, GJA1, HMGB1 (includes EG:3146), IL17RD, ITGB5, LDHA, MSH6, MYCN, OTX2, PLK2, REST, RHOB, SERPINE2, SERPINI1, SFRP2, SGK1, SIVA1, SLC2A1, SLC2A3, SMAD2, SMARCA2, SPRY2, TJP2, TSC1 | 46 |
| Cell Death | apoptosis | 6.37E-03 | BARD1, BLNK, CADM1, CCND1, CCND2, CTBP2, CTH, CXCL12, CXCR4, DHCR24, DUSP4, DUSP6, EZR, GFRA1, IL17RD, ITGB5, LDHA, MYCN, OTX2, PLK2, REST, RHOB, SERPINE2, SFRP2, SGK1, SIVA1, SLC2A1, SMAD2, SMARCA2, SPRY2, TSC1 | 31 |
| Cellular Development | differentiation | 7.98E-04 | ANXA2, BLNK, CA2, CCND1, CCND2, CD38, CNTN4, CXCL12, CXCR4, DHCR7, ELAVL2, EOMES, FECH, GFRA1, HMGB1 (includes EG:3146), IFRD1, IL17RD, ITGB5, KLF1, MRAS, MYCN, NLGN1, OTX2, PITX2, REST, SFRP2, SMAD2, SPRY1, SPRY2, TSC1 | 30 |
| Cellular growth and proliferation | proliferation | 1.53E-03 | AGK, ANXA2, B3GNT2, BARD1, BLNK, BUB1, CADM1, CCND1, CCND2, CD38, CDC37, CTBP2, CTH, CXCL12, CXCR4, DHCR7, DHCR24, FDFT1, FGF13, GJA1, HMGB1 (includes EG:3146), ITGB5, LAMA1, LDHA, MYCN, NRIP1, OTX2, PDGFA, PITX2, RARRES1, RHOB, SERPINE2, SMAD2, SMARCA2, SPRY1, SPRY2, TJP2, TMEFF2, TRIM24, TSC1 | 40 |
| Embryonic Development | developmental process | 8.14E-04 | CXCL12, CXCR4, EOMES, FDFT1, GJA1, LAMA1, OTX2, PITX2, SFRP2, SMAD2, SPRY1, TSC1 | 12 |
| Molecular Transport | mobilization | 1.99E-02 | BLNK, CXCL12, CXCR4, GNAI1, PPAP2A | 5 |
| Organismal Development | developmental process | 6.21E-04 | BARD1, C16ORF80, CADM1, CCND1, CCND2, CTBP2, CXCL12, EOMES, FDFT1, GJA1, HMGB1 (includes EG:3146), KLF1, LAMA1, MRAS, OTX2, PDGFA, PITX2, PLK2, SFRP2, SLC34A2, SMAD2, TJP2, TRIM24, TSC1 | 24 |
