# Supplementary material for: Basic Fibroblast Growth Factor Activates MEK/ERK Cell Signaling Pathway and Stimulates the Proliferation of Chicken Primordial Germ Cells
Source: PLoS One. 2010 Sep 23;5(9):e12968. doi: 10.1371/journal.pone.0012968 (PMC2944891; doi:10.1371/journal.pone.0012968)
Supplement: Table S4 — Regulation of cell proliferation and survival genes by bFGF. aFrom microarray data from three samples of cultured PGCs after bFGF withdrawal. bFrom microarray data from three samples of cultured PGCs after bFGF replacement. (0.09 MB DOC) [file pone.0012968.s004.doc]

| Gene | Description | Fold change  (–bFGF)^a^ | Fold change  (+bFGF)^b^ | Accession No. | Functional Category |
| --- | --- | --- | --- | --- | --- |
| AGK | acylglycerol kinase | –1.43 | 1.50 | BX271909 | Proliferation |
| ANXA2 | annexin A2 | 1.50 | –1.28 | NM_205351.1 | Proliferation |
| B3GNT2 | UDP-GlcNAc | 1.28 | –1.28 | CR353032.1 | Proliferation |
| BARD1 | BRCA1 associated RING domain 1 | –1.49 | 1.44 | ENSGALT00000005512.1 | Proliferation |
| CD38 | CD38 molecule | 1.68 | –1.68 | BU262827 | Proliferation |
| CXCR4 | chemokine (C-X-C motif) receptor 4 | –1.41 | 1.45 | AF294794.1 | Proliferation |
| FGF13 | fibroblast growth factor 13 | –1.59 | 1.59 | BU339287 | Proliferation |
| GJA1 | gap junction protein, alpha 1, 43 kDa | –2.48 | 2.84 | NM_204586.1 | Proliferation |
| ITGB5 | integrin, beta 5 | –1.73 | 2.09 | NM_204483.1 | Proliferation |
| LAMA1 | laminin, alpha 1 | 1.44 | –1.61 | ENSGALT00000023781.1 | Proliferation |
| OTX2 | orthodenticle homeobox 2 | –1.88 | 1.58 | NM_204520.1 | Proliferation |
| PDGFA | platelet-derived growth factor alpha polypeptide | 2.96 | –3.53 | AB031021.1 | Proliferation |
| RHOB | ras homolog gene family, member B | –1.92 | 2.44 | NM_204909.1 | Proliferation |
| SERPINE2 | serpin peptidase inhibitor, clade E (nexin, plasminogen activator inhibitor type 1), member 2 | –1.43 | 1.40 | CR352494.1 | Proliferation |
| SMAD2 | SMAD family member 2 | –1.42 | 1.82 | NM_204561.1 | Proliferation |
| SMARCA2 | SWI/SNF related, matrix associated, actin-dependent regulator of chromatin, subfamily a, member 2 | 1.65 | –1.84 | NM_205139.1 | Proliferation |
| SPRY2 | sprouty homolog 2 (Drosophila) | –2.92 | 4.05 | BU137030 | Proliferation |
| TJP2 | tight junction protein 2 (zona occludens 2) | –1.28 | 1.42 | NM_204918.1 | Proliferation |
| TMEFF2 | transmembrane protein with EGF-like and two follistatin-like domains 2 | –2.30 | 2.76 | BX933949.1 | Proliferation |
| CCND1 | cyclin D1 | –1.49 | 1.83 | ENSGALT00000012216.1 | Cell cycle |
| PPAP2A | Phosphatidic acid phosphatase type 2A | –2.64 | 3.55 | BX934014.2 | Cell cycle |
| SGK1 | serum/glucocorticoid regulated kinase 1 | –2.49 | 4.29 | NM_204476.1 | Cell cycle |
| AKAP12 | A kinase (PRKA) anchor protein (gravin) 12 | 1.52 | –1.43 | BU127250 | Cell death |
| ATP2B1 | ATPase, Ca++ transporting, plasma membrane 1 | –1.39 | 1.32 | BU293343 | Cell death |
| CD82 | CD82 molecule | 1.40 | –1.55 | AJ719947 | Cell death |
| EAF2 | ELL associated factor 2 | 1.28 | –1.66 | CR406825.1 | Cell death |
| SLC2A3 | solute carrier family 2 (facilitated glucose transporter), member 3 | –1.61 | 1.81 | NM_205511.1 | Cell death |
| DUSP4 | dual specificity phosphatase 4 | –1.53 | 1.88 | NM_204838.1 | Apoptosis |
| DUSP6 | dual specificity phosphatase 6 | –3.32 | 3.96 | NM_204354.1 | Apoptosis |
| IL17RD | interleukin 17 receptor D | –4.04 | 5.67 | NM_204515.1 | Apoptosis |
| SLC2A1 | solute carrier family 2 (facilitated glucose transporter), member 1 | –1.87 | 1.41 | NM_205209.1 | Apoptosis |
