# Supplementary material for: Basic Fibroblast Growth Factor Activates MEK/ERK Cell Signaling Pathway and Stimulates the Proliferation of Chicken Primordial Germ Cells
Source: PLoS One. 2010 Sep 23;5(9):e12968. doi: 10.1371/journal.pone.0012968 (PMC2944891; doi:10.1371/journal.pone.0012968)
Supplement: Table S5 — Information of the primer sets used for RT-PCR analysis. (0.03 MB DOC) [file pone.0012968.s005.doc]

| Gene | Primer sequence | Product size (bp) |
| --- | --- | --- |
| *NANOG* | CAGCAGACCTCTCCTTGACC  AAGCCCTCATCCTCCACAGC | 586 |
| *POUV* | GCCAAGGACCTCAAGCACAA  ATGTCACTGGGATGGGCAGA | 511 |
| *CVH* | GGGAAGATCAGTTTGGTGGA  GACAAAGAAAGGCTGCAAGG | 388 |
| *DAZL* | CGTCAACAACCTGCCAAGGA  TTCTTTGCTCCCCAGGAACC | 540 |
| *KIT* | GTGGGCAAGAAGTGGAAGCC  GCAAACCAAGCATCTCATCCC | 239 |
| *GAPDH* | CACAGCCACACAGAAGACGG  CCATCAAGTCCACAACACGG | 443 |
