# Supplementary material for: Basic Fibroblast Growth Factor Activates MEK/ERK Cell Signaling Pathway and Stimulates the Proliferation of Chicken Primordial Germ Cells
Source: PLoS One. 2010 Sep 23;5(9):e12968. doi: 10.1371/journal.pone.0012968 (PMC2944891; doi:10.1371/journal.pone.0012968)
Supplement: Table S6 — Information of the primer sets used for quantitative RT-PCR analysis. (0.04 MB DOC) [file pone.0012968.s006.doc]

| Gene | Primer sequence |
| --- | --- |
| *NANOG* | CAGCAGACCTCTCCTTGACC  TTCCTTGTCCCACTCTCACC |
| *POUV* | GTTGTCCGGGTCTGGTTCT  GTGGAAAGGTGGCATGTAGAC |
| *CVH* | TGACTTATGTCCCCCCTCCT  GTAATGGTGCTGGAGGGTCA |
| *DAZL* | TCCCAGAGCCCACACAGATG  AAGTGATGCGCCCTCCTCTC |
| *SPRY2* | GGGAAGTGCAAGTGCAAGGA  GACTGACTGCAAGAGCAGGG |
| *PPAP2A* | CCAGCCTGACTGGACACAAA  ACAAGTCTCGCCCAGTCTCC |
| *GJA1* | TGGACTGTTTCCTGTCCCGT  TCCTTGGAAGGGCTCATCGT |
| *TMEFF2* | GCTCCAACGGTGACACCTAC  GCCTTCATTGACTCCGTCCC |
| *IL17RD* | CCGTGGGCTGGACCAATAAG  GGCGAAGTCTTTCCACAGGG |
| *DUSP6* | TACTTCACCACCCCTGCCAA  CAGTGCCACAGGCCAGAAAA |
| *SGK1* | GCTTTATGGCCTGCCACCTT  CCTTGGCACCAAGCCTCTTT |
| *ITGB5* | TTGGTTTCCGCCACCTCTTG  GCCAGCCGATCTTCTCCTTG |
| *GAPDH* | ACACAGAAGACGGTGGATGG  GGCAGGTCAGGTCAACAACA |
